# Acacetin ameliorates insulin resistance in obesity mice through regulating Treg/Th17 balance via MiR-23b-3p/NEU1 Axis

**DOI:** 10.1186/s12902-021-00688-8

**Published:** 2021-03-29

**Authors:** Yan Wei, Jianhong Jing, Zhiping Peng, Xiaoqian Liu, Xueyang Wang

**Affiliations:** 1 Department of Endocrinology and Metabolism, Hangzhou TCM Hospital Affiliated to Zhejiang Chinese Medical University, No.453 Stadium Road, Xihu District, Zhejiang, 310007 Hangzhou China; 2 Department of Geriatrics, Hangzhou TCM Hospital Affiliated to Zhejiang Chinese Medical University, No.453 Stadium Road, Xihu District, Zhejiang, 310007 Hangzhou China

**Keywords:** Acacetin, Insulin resistance, Inflammation, MiR-23b-3p, Neuraminidase 1, Obesity, T cells/T helper 17 cells

## Abstract

**Background:**

The role of miR-23b-3p in insulin resistance (IR) remained poorly understood.

**Methods:**

After acacetin injection, obesity-induced IR model was constructed with or without miR-23b-3p upregulation and Neuraminidase 1 (NEU1) overexpression in mice. Body weight, serum metabolite and fat percent of the mice were measured. Tests on oral glucose and insulin tolerance were performed, and inflammatory cytokines C-reactive protein (CRP), Interleukin-6 (IL-6), tumor necrosis factor-α (TNF-α), and monocyte chemoattractant protein 1 (MCP1) levels were quantified with enzyme-linked immunosorbent assay (ELISA). The binding sites between miR-23b-3p and NEU1 were predicted by TargetScan, and verified using dual-luciferase reporter assay. Relative expressions were detected with quantitative real-time polymerase chain reaction (qRT-PCR) and Western blot. Proportion of Treg and Th17 cells in total CD4^+^ T cells was detected with flow cytometry.

**Results:**

MiR-23b-3p offset the effects of acacetin on body weight, fat percent, inflammatory cytokines levels and expressions of markers of regulatory T cells (Treg cells) and T helper 17 cells (Th17 cells), NEU1 and miR-23b-3p. NEU1 was a target of miR-23b-3p, and overexpressed NEU1 reversed the effects of upregulated miR-23b-3p on reducing Treg cells but increased body weight, fat percent and inflammatory cytokines levels, percentage of Th17 cells, and upregulated NEU1 expression.

**Conclusion:**

Upregulation of miR-23b-3p offset the effects of acacetin on obesity-induced IR through regulating Treg/Th17 cell balance via targeting NEU1.The present findings provide a possible prevention strategy for obesity-induced IR.

**Supplementary Information:**

The online version contains supplementary material available at 10.1186/s12902-021-00688-8.

## Background

Over the last decades, the incidence of obesity has increased significantly [1]. Obesity with excessive accumulation of white adipose tissues will lead to a series of complications such as systematic insulin resistance (IR) [2]. IR is defined as the disturbance on glucose regulation and is characterized by a high insulin level [3]. Obesity could induce constant secretion of hormones and cytokines in adipose tissues, thereby causing low-grade local inflammation and local-grade inflammation. Study also indicated that investigating the mechanisms of local low-grade inflammation is fundamental to the prevention against obesity-induced IR [4].

MicroRNAs (miRNAs; miRs) are 18–25 nucleotides long RNA and have modulatory effects on gene expressions by interacting with mRNA targets through binding to the 3′-untranslated regions (3′-UTRs) [5]. Growing evidence indicated that certain miRNAs are implicated in obesity-induced IR. As Gallo et al. showed that miR-483-5p is associated with obesity and cardiovascular diseases, and is also correlated with body mass index (BMI), fasting insulin (FI), high-density lipoprotein (HDL) and triglycerides [6]. Genetic ablation of miR-33 could increase pre-adipocyte proliferation, enhance lipid uptake and impair lipolysis [7]. In addition, miR-29a expression is upregulated in obese adipose tissues (ATMs)-derived exosomes and could transfer into adipocytes, myocytes and hepatocytes, eventually leading to IR [8]. MiR-23b-3p is a member of miR-23 family, and its detailed role and biological functions in obesity-induced IR remained to be fully addressed.

Neuraminidases (NEUs) are involved in regulating metabolic pathways, including glucose homeostasis [9]. NEUs could regulate molecular and cellular recognition events with sialyltransferases, and catalyze the removal of sialic acid from glycoproteins, oligosaccharides and sialylated glycolipids [9]. NEU1, in particular, modulates cellular receptors involved in activation or inhibition of various signaling pathways by cleaving sialic acids on the glycan chains in the receptors to reverse the process of IR [9–11]. Chang et al. pointed out that NEU1 is the target of miR-125b to suppress the progression of gastric cancer [12]. However, relationship between miR-23b-3p and NEU1 in obesity-induced IR is less discussed. In addition, acacetin has suppressive effects on adipogenesis and could attenuate lipid accumulation in mice with obesity [13]. However, its efficacy in obesity-induced IR remained poorly understood. We set out to uncover the roles and biological functions of miR-23b-3p and NEU1 in obesity-induced IR, hoping to find a possible prevention against obesity-induced IR in clinical practice.

## Materials and methods

### Ethics statement

All animal experiments were conduction in compliance with the principles of China Council on Animal Care and Use and performed in Hangzhou Hospital of Traditional Chinese Medicine. The research has been recommended by the Ethic Committee of Experimental Animals of Hangzhou Hospital of Traditional Chinese Medicine (approval number: NFMK-2019092401). Every effort to minimize animal pain and discomfort were taken into account.

### Animal model establishment

Animal model was constructed as previously reported [14]. In our research, acacetin (catalog no. 00017) was purchased from Sigma-Aldrich (St Louis, MO). Male C57BL/6 J mice (stock no. 000664; total number = 120, 6 weeks old, 20 ± 2 g) were commercially acquired from Jackson Laboratory (Bar Harbor, ME) and kept in non-pathogen individual cages with 12 h (h) light/dark cycles at 21–25 °C in 50–60% humidity.

After a week adaptation period upon arrival, 50 of the total mice were assigned into five groups (*n* = 10 for each group) as following: Control, Model, Acacetin, Acacetin+ MC, Acacetin+MC groups.

Control group: mice were given normal diet ([10% Kcal fat, 3.82 Kcal/g], D12450B, Research Diets, New Brunswick, NJ);

Model group: mice were given high-fat diet ([60% Kcal fat, 5.21Kcal/g], D12492, Research Diets);

Acacetin group: mice were given high-fat diet and then subjected to intraperitoneal injection of 10 mg/kg acacetin;

Acacetin+mimic control (MC) group: mice were given high-fat diets and then subjected to intraperitoneal injection of 10 mg/kg acacetin, followed by tail-vein injection of miR-23b-3p MC lentivirus carrier;

Acacetin+mimic (M) group: mice were first fed with high-fat diets and then given intraperitoneal injection of 10 mg/kg acacetin, followed by tail-vein injection of miR-23b-3p lentivirus carrier for 8 weeks. Fat percent of mice was determined under Faxitron animal bone densitometer (UltraFocus, Tucson, AZ).

Other mice (*n* = 70) were divided into the following seven groups (*n* = 10 for each group): Control, Model, MC, M, NC, NEU1, M + NEU1 groups.

Control and model groups: mice were processed as described above;

MC group: mice were first given high-fat diet and then received tail-vein injection of miR-23b-3p MC lentivirus carrier;

M group: mice were first given high-fat diet and then received tail-vein injection of miR-23b-3p lentivirus carrier;

Negative control (NC) group: mice were first given high-fat diet and then received tail-vein injection of lentivirus carrier of NC for NEU1;

NEU1 group: mice were first given high-fat diet and then received tail-vein injection of lentivirus carrier of NEU1 overexpression plasmid;

M + NEU1 group: mice were first given high-fat diet and then received tail-vein injection of lentivirus carrier of miR-23b-3p and NEU1 overexpression plasmid. For the experiment, lentivirus carriers were synthesized and obtained from Thermo Fisher Scientific (Waltham, MA).

Finally, as previously described, all the mice were sacrificed after anesthesia using Ketamine (80 mg/kg, K2753, Sigma-Aldrich), xylazine (8 mg/kg, X1126, Sigma-Aldrich) and acepromazine (0.5 mg/kg, A7111, Sigma-Aldrich) [15]. The spleen and blood were collected and stored at 4 °C for subsequent studies.

### Serum metabolite measurements

For measurements on fasting blood glucose (FBG) and FI levels, the mice were first given an intraperitoneal injection of acacetin (10 mg/kg) and D-glucose (2 g/kg mice body weight; G8270, Sigma-Aldrich). Then, the blood samples were collected, and FBG level was measured by Glucose Monoreagent Kit (K082–3, Bioclin, Belo Horizonte, Brazil) in a glucometer (Accu-Chek, Roche Diagnostics, Rotkreuz, Switzerland), while FI level was quantified with insulin mouse kit for enzyme-linked immunosorbent assay (ELISA) (EMINSX5; Invitrogen, Carlsbad, CA).

### Oral glucose tolerance test (OGTT) and insulin tolerance test (ITT)

After intraperitoneal injection of acacetin, oral glucose tolerance test (OGTT) and insulin tolerance test (ITT) were respectively carried out in line with a previous description [16].

For OGTT, the mice with miR-23b-3p mimic and NEU1 overexpression were fasted overnight for 12 h before each experiment. A tail cut (1–2 mm) was performed with a sterilized scissor (FS001; Beyotime, Shanghai, China), and around 30 μL of blood sample was collected using fresh capillary tube (15401–560, VWR, Atlanta, GA) for quantifying basal blood glucose level (= time point 0) with a glucometer (Roche Diagnostics, Switzerland). 15 min (min) after oral administration of D-glucose (2 g/kg mice body weight, Sigma-Aldrich), about 30 μL blood was collected to measure blood glucose level.

For ITT, mice with miR-23b-3p mimic and NEU1 overexpression were fasted overnight for 12 h before each experiment, and basal blood glucose level was measured at 0 min. Insulin (0.5 U/kg mice body weight, I5500, Sigma-Aldrich) was intraperitoneally injected into the mice, and whole blood glucose level was measured by a glucometer (Roche Diagnostics, Switzerland) after 15 min.

### Enzyme-linked immunosorbent assay (ELISA)

The levels of inflammatory cytokines C-reactive protein (CRP, catalog no. EM20RB, Invitrogen), Interleukin-6 (IL-6, catalog no. BMS603–2, Invitrogen), tumor necrosis factor-α (TNF-α, catalog no. BMS607–3, Invitrogen), and monocyte chemoattractant protein 1 (MCP1, catalog no. BMS6005, Invitrogen) in serum of the mice were quantified with their specific ELISA kits. OD value was detected using automated ELISA systems (11,050,010; Titertek-Berthold, Pforzheim, Germany).

### Isolation of spleen cells

Mouse spleen cells were isolated following a previous description [17]. In detail, the spleen was first perfused in 10 mL phosphate buffered saline (PBS), which was then injected into left ventricular before dissection. Then the spleen was cut into a homogenous paste on the dish plate using scalpel, and treated with 1 mL of enzyme cocktail containing 1 mg/mL Collagenase D (C0130, Sigma-Aldrich), 100 μg/mL Deoxyribonuclease I (DNase I, D5025, Sigma-Aldrich) and 0.6 U/mL Dispase (#07923, Stemcell, Tokyo, Japan) in Dulbecco’s modified eagle’s medium (DMEM, D5030, Sigma-Aldrich) with 2% fetal bovine serum (FBS, F2442, Sigma-Aldrich). For mechanical grinding, the spleen was ground to collect spleen cells. Following incubation in 24-well plates at 37 °C for 30 min, cell suspension was passed through a 100 μm Falcon nylon cell strainer (352,360, Corning Inc., Corning, NY), and cells were resuspended in DMEM with 10% FBS and 5 mM (EDTA (E6758, Sigma-Aldrich).

### Flow cytometry

The isolated spleen cells from C57BL/6 J mice were subjected to flow cytometric analysis and then stained with the following fluorescence-conjugated antibodies: anti-Cluster of Differentiation 4 (CD4) antibody (FITC, ab218745, Abcam, Cambridge, UK), anti-CD25 antibody, (PE, 12–0251-82, Thermo Fisher Scientific), anti-Forkhead box P3 (Foxp3) antibody (Allophycocyanin (APC), ab200568, Abcam, UK) and anti-IL-17 antibody (Biotin, C48308-Biotin, Signalway Antibody, College Park, MD) at 4 °C for 1 h in the dark. The secondary antibodies included goat anti-rat IgG (Alexa Fluor® 488, ab150157, Abcam, UK) and goat anti-mouse IgG (Alexa Fluor® 647, ab150115, Abcam, UK). Then lymphocyte subsets in total CD4^+^ T cells were analyzed with flow cytometry in the dark. Changes on percentages of regulatory T cells (Treg cells, CD4^+^CD25^+^Foxp3^+^) and T helper 17 cells (Th17 cells, CD4^+^IL-17^+^) in total CD4^+^ T cells were measured with CytoFLEX Flow Cytometer (B96622, Beckman Coulter, Indianapolis, IN) in Kaluza C Analysis Software (Beckman Coulter).

### Cell culture

HEK-293 T cell line (catalog no. CRL-11268) was purchased from American Type Culture Collection (ATCC; Manassas, VA) and cultured in high-glucose DMEM (#90013, Solarbio, China) plus 10% FBS and 1% penicillin/streptomycin (P1400, Solarbio, China).

### Bioinformatics and dual-luciferase reporter assay

The binding sites between miR-23b-3p and NEU1 were predicted by TargetScan, and then dual-luciferase reporter assay was employed for confirmation.

NEU1 3′-UTR containing miR-23b-3p target sites obtained from Gene Pharma (Shanghai, China)was ligated into luciferase vector pMirGLO (AM5795, Thermo Fisher Scientific) to form reporter plasmids of wild-type NEU1 (NEU1-WT). QuikChange II Site-directed Mutagenesis Kit (200,523, Agilent, Santa Clara, CA) was used to perform 3′-UTR mutagenesis to create reporter plasmids of mutated NEU1 (NEU1-MUT). Then, for dual-luciferase reporter assay, we first cultured HEK-293 T cells in 96-well plate at an adjusted density of 5 × 10^3^ cells/well, and subsequently transfected 200 ng of miR-23b-3p mimic (M, sequence: 5′-AUCACAUUGCCAGGGAUUACCAC-3′; B02003, Gene Pharma, China), its control (MC, sequence: 5′-AUCAUAGGUCUCAUGGCCAACAC-3′; B04001, Gene Pharma, China), and 50 ng recombinant reporter plasmids of NEU1-WT (sequence: 5′-CUGUAGAAUUGAAUCAAUGUGAA-3′) and NEU1-MUT (sequence: 5′-CUGUAGAAUUGAAUCCGACCAUA-3′) into the cells using Lipofectamine 3000 reagent (L3000–001, Invitrogen). Following 48 h, HEK-293 T cells were harvested, and its luciferase activity was measured with dual-luciferase reporter assay system (E1910; Promega, Madison, MI). *Renilla* luciferase activity was used for firefly luciferase activity normalization.

### RNA isolation and quantitative real-time polymerase chain reaction (qRT-PCR)

Total RNA was isolated by Trizol (15596–018, Invitrogen). The RNA concentration was determined by a Nano Drop 2000 spectrometer (Thermo Fisher Scientific). CDNA was synthesized from 1 μg of total RNA by a RevertAid H Minus II First-Strand cDNA Synthesis Kit (K1631, Thermo Fisher Scientific). QPCR was performed with One-step PrimeScript RT-PCR kit (RR064B, Takara, Shiga, Japan) in Touch real-time PCR Detection system (CFX96, Bio-Rad) under the following conditions: at 95 °C for 5 min, and 40 cycles at 95 °C for 5 s (s), and at 60 °C for 30 s. U6 and GAPDH were internal controls. Sequences for primer were listed in Table 1. Relative gene levels were quantified by 2^-ΔΔCT^ method [18].

**Table 1:** List of antibodies used for western blots

Protein	Host species	Catalog Number	Company	Antibody Dilution
TGF-β1	Rabbit	ab92486	Abcam	1:10000
IL10	Rat	ab189392	Abcam	1:1000
IL-17	Rabbit	ab79056	Abcam	1:500
IL-6	Rabbit,	ab208113	Abcam	1:10000
NEU1	Rabbit	ab233119	Abcam	1:2000
GAPDH	Rabbit	ab181602	Abcam	1:10000

### Western blot

Protein expressions of Treg and Th17 markers (Transforming growth factor-β1, TGF-β1; Interleukin-10, IL-10; IL-17 and IL-6) and NEU1 were measured with Western blot [19]. The total protein was lysed and extracted from the harvested HEK-293 T cells using RIPA buffer (sc-24,948, Santa Cruz Biotech, Dallas, TX). Bicinchoninic acid (BCA) protein kit (K813; BioVision, Milpitas, CA) was applied to quantify the protein concentration. 20 μg protein sample lysates were first electrophoresed with sodium dodecyl sulfate-polyacrylamide gel electrophoresis (SDS-PAGE; P1200; Solarbio, China), and then moved onto diluted polyvinylidene fluoride (PVDF) membrane (YA1701, Solarbio, China). Subsequently, the membrane was blocked using 5% skimmed milk for 2 h, and then was incubated in the primary antibodies (Table 1) overnight at 4 °C. GAPDH was an internal control. After that, the membrane was washed with tris-buffer saline tween (TBST) for three times and treated with the secondary antibodies (goat anti-rabbit IgG H&L (1:10000, sc-2004, Santa Cruz Biotech) and goat anti-rat IgG H&L (ab97057, 1:2000, Abcam, UK)) at room temperature for 1 h. Protein strip was developed with enhanced chemiluminescence (ECL) kit (SW2020; Solarbio, China). Grey values of the strips were further calculated under the calculation of ImageJ 5.0 (Bio-Rad, Hercules, CA) in iBright CL750 Imaging System (A44116; Invitrogen). The original, unprocessed gel/blot images are included in additional file 1.

### Statistical analysis

All the experiments were independently performed over three times. The data were expressed as mean ± standard deviation (SD). Statistics were analyzed using SPSS 21.0 (SPSS, Chicago, IL). Statistics difference were determined with student’s *t* test and one-way ANOVA with post hoc test Dunnett’s. Significant difference was considered when *P*-value < 0.05.

## Results

### MiR-23b-3p upregulation abrogated the inhibitory effects of acacetin on IR and levels of inflammatory cytokines in obesity mice

**Fig. 1:**
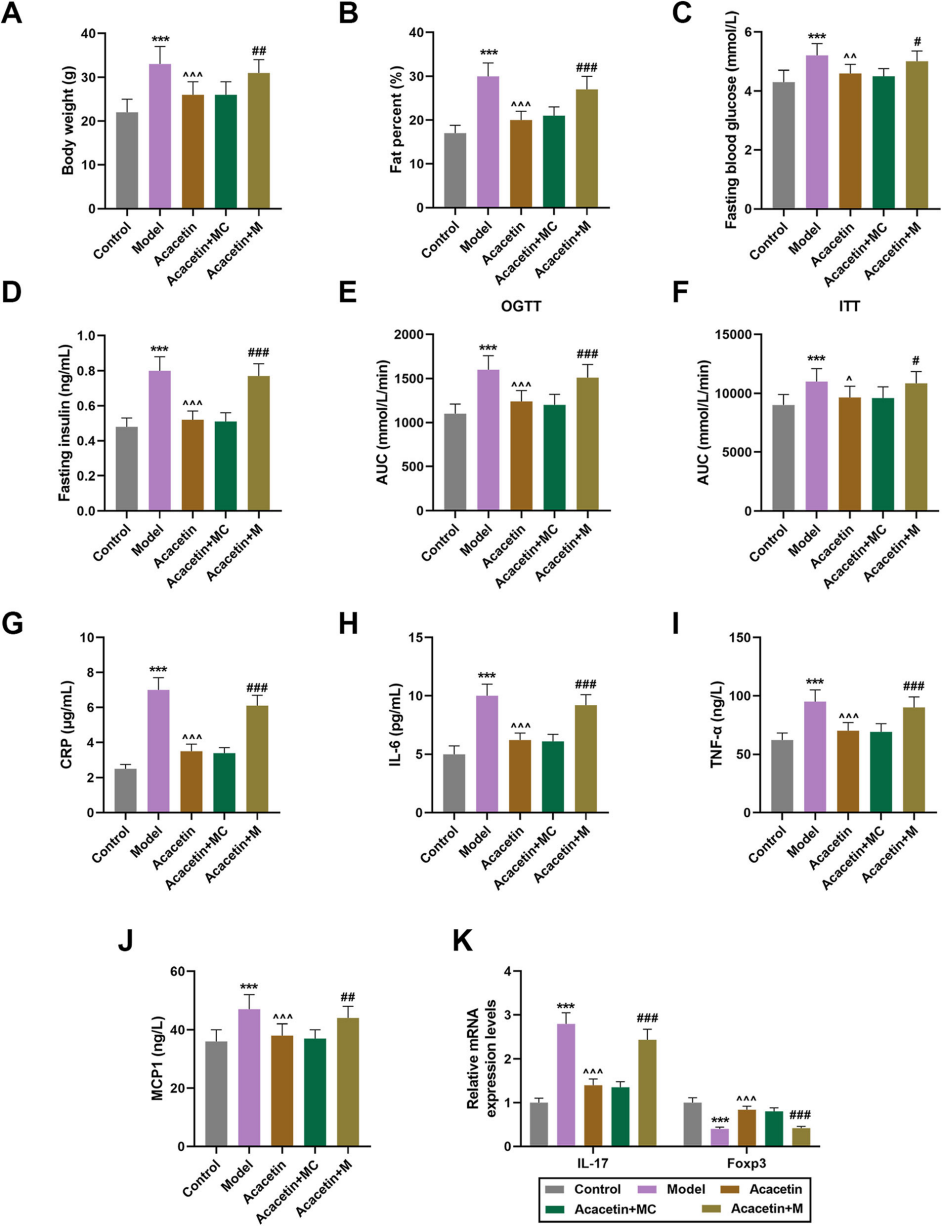
MiR-23b-3p upregulation promoted the effects of obesity-induced IR of mice. **a** Body weight of mice after obesity-induced IR model construction, Acacetin injection and miR-23b-3p upregulation was measured. **b** Fat percent of mice was calculated after obesity-induced IR model construction, acacetin injection and miR-23b-3p upregulation. **c-d** Levels of fasting blood glucose (**c**) and fasting insulin (**d**) after obesity-induced IR model construction, acacetin injection and miR-23b-3p upregulation was determined. **e-f** AUC of mice for OGTT (**e**) and ITT (**f**) after obesity-induced IR model construction, acacetin injection and miR-23b-3p upregulation were quantified. **g-j** Levels of inflammatory cytokines CRP (**g**), IL-6 (**h**), TNF-α (**i**) and MCP1 (**j**) after obesity-induced IR model construction, acacetin injection and miR-23b-3p upregulation were detected with ELISA. **k** Relative expressions of IL-17 and Foxp3 after obesity-induced IR model construction, acacetin injection and miR-23b-3p upregulation were determined using qRT-PCR. GAPDH was used as internal control. All experiments have been performed in triplicate and data were expressed as mean ± standard deviation (SD). ^*^P < 0.05, ^**^P < 0.01, ^***^P < 0.001, vs. Control; ^^^*P* < 0.05, ^^^^*P* < 0.01, ^^^^^*P* < 0.001, vs. Model; ^#^*P* < 0.05, ^##^*P* < 0.01, ^###^*P* < 0.001, vs. Acacetin+mimic control (MC). miR-23b-3p: MicroRNA-23b-3p; AUC: Area under the Curve; OGTT: Oral glucose tolerance test; ITT: Insulin tolerance test; CRP: C-reactive protein; IL-6: Interleukin-6; TNF-α: tumor necrosis factor-α; MCP1: monocyte chemoattractant protein 1; ELISA: enzyme-linked immunosorbent assay; Foxp3: Forkhead Box P3; qRT-PCR: quantitative real-time polymerase chain reaction

To determine the role of miR-23b-3p, we constructed an obesity-induced IR model in mice through acacetin injection with or without the presence of miR-23b-3p mimic, and then measured body weight and fat percent of the mice. In Fig. 1a-b, we found that both body weight and fat percent were increased in mice after the model construction, while acacetin injection reduced body weight and fat percent (Fig. 1a-b, *P* < 0.001). Also, after upregulating miR-23b-3p, mice with acacetin injection showed a higher body weight and fat percent as compared with MC (Fig. 1a-b, *P* < 0.01), suggesting that miR-23b-3p could reverse the effects of acacetin on body weight and fat percent in the mice.

We also found that FBG and FI were upregulated after obesity-induced IR model construction, but were downregulated by acacetin injection (Fig. 1c-d, *P* < 0.01). MiR-23b-3p upregulation in acacetin-injected mice promoted the levels of FBG and FI (Fig. 1c-d, *P* < 0.05). Then, to confirm that obesity-induced IR model was successfully established, glucose and insulin tolerance tests were performed. As shown in Fig. 1e-f, area under the curve (AUC) of both OGTT and ITT was increased after model construction in comparison with Control, but lower AUC of OGTT and ITT was found after acacetin injection (Fig. 1e-f, *P* < 0.05). AUC of OGTT and ITT was increased after miR-23b-3p upregulation in obesity-induced IR mice with injection of acacetin (Fig. 1e-f, *P* < 0.05).

The levels of inflammatory cytokines (CRP, IL-6, TNF-α, and MCP1) were measured with ELISA. In Fig. 1g-j, we found that the levels of inflammatory cytokines were upregulated after obesity-induced IR model construction, whereas acacetin resulted in opposite results (Fig. 1g-j, *P* < 0.01). Also, upregulating miR-23b-3p abrogated the effects of acacetin on inflammatory cytokines levels (Fig. 1g-j, *P* < 0.05).

### MiR-23b-3p upregulation abrogated the effects of acacetin on marker expressions of Th17 and Treg cells

We measured expressions of Th17 (Interleukin-17, IL-17) and Treg cell markers (Forkhead box p3, Foxp3) using qRT-PCR. In Fig. 1k, IL-17 expression was upregulated but Foxp3 expression was downregulated after obesity-induced IR model construction, whereas acacetin injection downregulated IL-17 expression and upregulated Foxp3 expression (Fig. 1k, *P* < 0.001). Meanwhile, we also found that upregulating miR-23b-3p reversed the effects of acacetin on IL-17 and Foxp3 expressions (Fig. 1k, *P* < 0.001).

### NEU1 was the target of miR-23b-3p

**Fig. 2:**
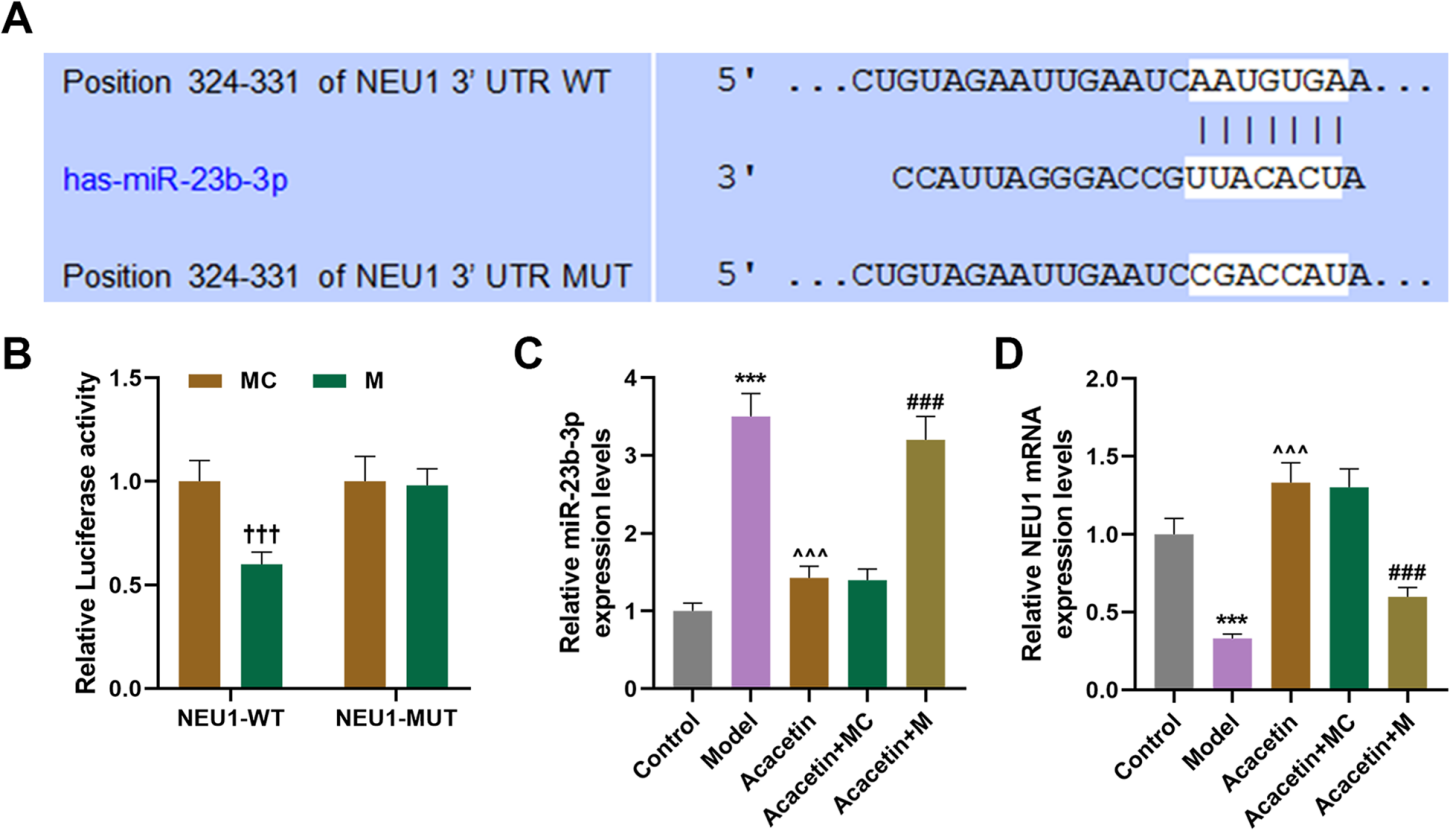
NEU1 was the target of miR-23b-3p, and miR-23b-3p upregulation further enhanced the effects of obesity on NEU1 expression. **a** Sequences of NEU1-WT (top), miR-23b-3p (middle) and NEU1-MUT (below) were listed. **b** Dual-luciferase reporter assay confirmed that NEU1 was the target of miR-23b-3p. **c-d** Relative NEU1 protein/GAPDH expressions after obesity-induced IR model construction and miR-23b-3p upregulation were measured with Western blot. GAPDH was employed as internal control. All experiments have been performed in triplicate and data were expressed as mean ± standard deviation (SD). ^†††^*P* < 0.001, vs. Control; ^***^P < 0.001, vs. Control; ^^^^^*P* < 0.001, vs. Model; ^###^*P* < 0.001, vs. Acacetin+MC. WT: wild-type; MUT: mutated; M: mimic; NEU1: Neuraminidase 1

TargetScan successfully predicted NEU1 as the target of miR-23b-3p, and their complementary binding sites were presented in Fig. 2a. For confirmation, dual-luciferase reporter assay was conducted. In Fig. 2b, we found that compared with NEU1-WT-MC group, luciferase activity in NEU1-WT-M group was reduced (Fig. 2b, *P* < 0.001), while that in NEU1-MUT-M group was not changed relative to NEU1-MUT-MC group, suggesting that NEU1 was a target of miR-23b-3p.

### MiR-23b-3p upregulation reversed the effects of acacetin on miR-23b-3p and NEU1 expressions in the mice with obesity-induced IR

To examine the possible correlation between NEU1 and miR-23b-3p, NEU1 protein expressions were measured after obesity-induced IR model construction, acacetin injection, and miR-23b-3p upregulation. In Fig. 2c-d, the results showed that miR-23b-3p expression was upregulated and NEU1 expression was downregulated after the model construction (Fig. 2c-d, *P* < 0.001). However, acacetin injection downregulated miR-23b-3p expression and upregulated NEU1 expression (Fig. 2c-d, *P* < 0.001). Furthermore, upregulated miR-23b-3p abrogated the effects of acacetin on miR-23b-3p and NEU1 expressions in mice with obesity-induced IR (Fig. 2c-d, *P* < 0.001).

### Overexpressed NEU1 reversed the effects of miR-23b-3p upregulation on percentage of Treg and Th17 cells

**Fig. 3:**
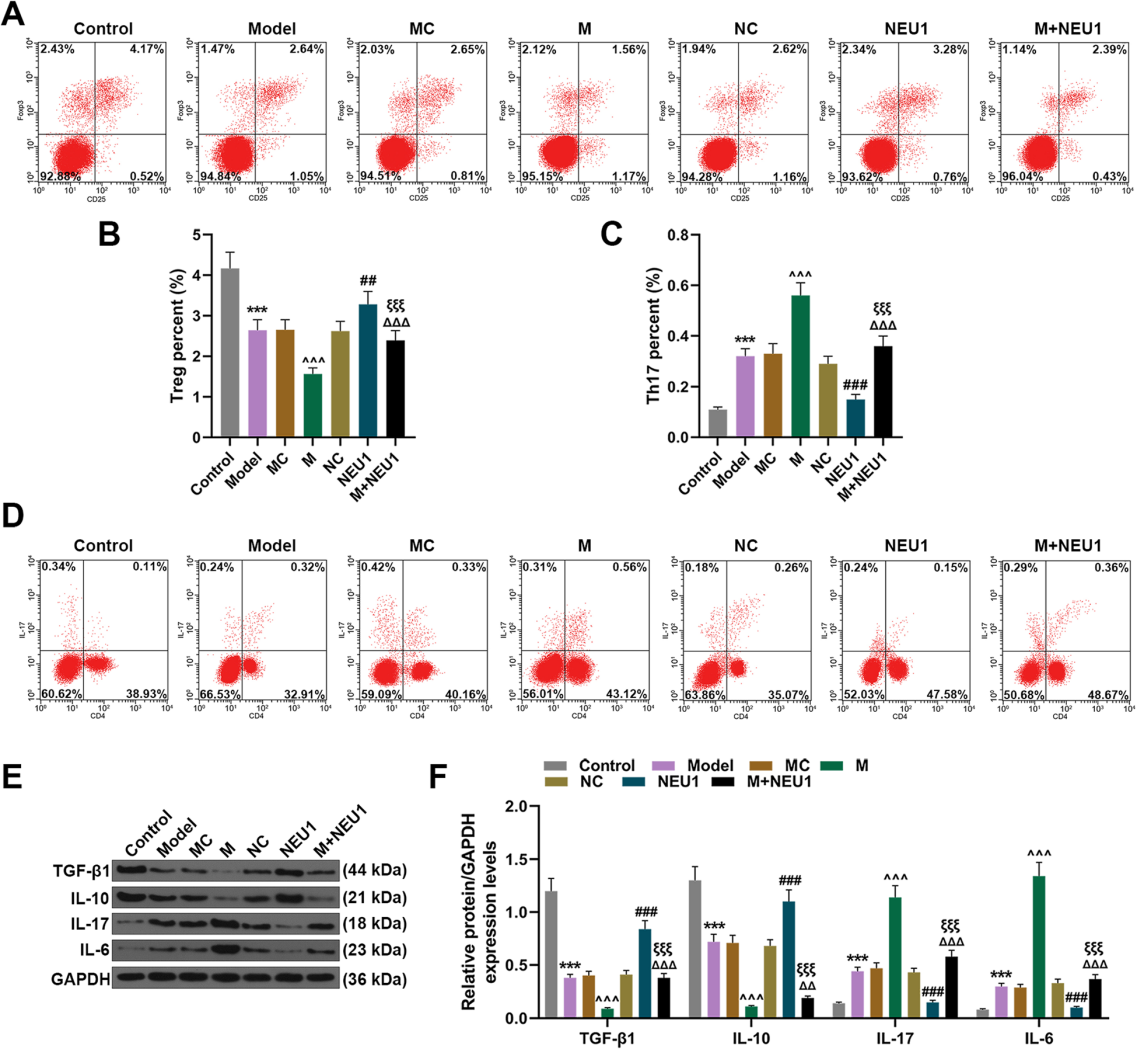
Overexpressed NEU1 reversed the effects of miR-23b-3p upregulation on Treg and Th17 cell percentage. **a-d** Percentages of Treg cells **(**CD4^+^ CD25^+^ Foxp3^+^**)** and Th17 cells **(**CD4^+^ IL-17^+^**)** after obesity-induced IR model construction and injection of lentivirus carriers for miR-23b-3p mimic and NEU1 overexpression plasmid were calculated by flow cytometry. **e-f** Protein/GAPDH expressions of TGF-β1, IL-10, IL-17 and IL-6 after obesity-induced IR model construction and injection of lentivirus carriers for miR-23b-3p mimic and NEU1 overexpression plasmid were measured with Western blot. GAPDH was employed as internal control. All experiments have been performed in triplicate and data were expressed as mean ± standard deviation (SD). ^***^P < 0.001, vs. Control; ^^^^^*P* < 0.001, vs. MC; ^##^*P* < 0.01, ^###^*P* < 0.001, vs. negative control (NC); ^ΔΔ^*P* < 0.01, ^ΔΔΔ^*P* < 0.001, vs. mimic (M); ^ξξξ^*P* < 0.001, vs. NEU1. Treg cells: regulatory T cells; Th17 cells: T helper 17 cells; TGF-β1: Transforming growth factor-β1

To examine the effects of miR-23b-3p and NEU1 on Treg/Th17 cells balance, after injection of lentivirus carrier of miR-23b-3p mimic and NEU1 overexpression plasmid, the percentages of Treg cells (CD4^+^CD25^+^Foxp3^+^) and Th17 (CD4^+^IL-17^+^) cells in total CD4^+^ T cells were determined with flow cytometry. As shown in Fig. 3a-d, it could be found that after obesity-induced IR model was established in mice, Treg cells was reduced and Th17 cells was increased in total CD4^+^ T cells (Fig. 3a-d, *P* < 0.001). Moreover, upregulating miR-23b-3p further enhanced the effects of obesity-induced IR on reducing Treg cells and increasing Th17 cells (Fig. 3a-d, *P* < 0.001). However, Treg cells were increased and Th17 cells were reduced following NEU1 overexpression, and NEU1 overexpression reversed the effects of miR-23b-3p on Treg and Th17 cells (Fig. 3a-d, *P* < 0.01).

Then, markers of Treg and Th17 cells were measured with Western blot. After an obesity-induced IR model in mice was constructed, both TGF-β1 and IL-10 protein expressions were downregulated but those of IL-17 and IL-6 were upregulated (Fig. 3e-f, *P* < 0.001). Upregulating miR-23b-3p further enhanced the effects of obesity-induced IR on downregulating TGF-β1 and IL-10 protein expressions and upregulating IL-17 and IL-6 expressions (Fig. 3e-f, *P* < 0.001 and additional file 1). Moreover, overexpressed NEU1 upregulated TGF-β1 and IL-10 protein expressions but downregulated IL-17 and IL-6 protein expressions, and reversed the effects of miR-23b-3p on TGF-β1, IL-10, IL-17 and IL-6 expressions (Fig. 3e-f, *P* < 0.001).

### Overexpressed NEU1 reversed the effects of miR-23b-3p upregulation on obesity-induced IR in mice

**Fig. 4:**
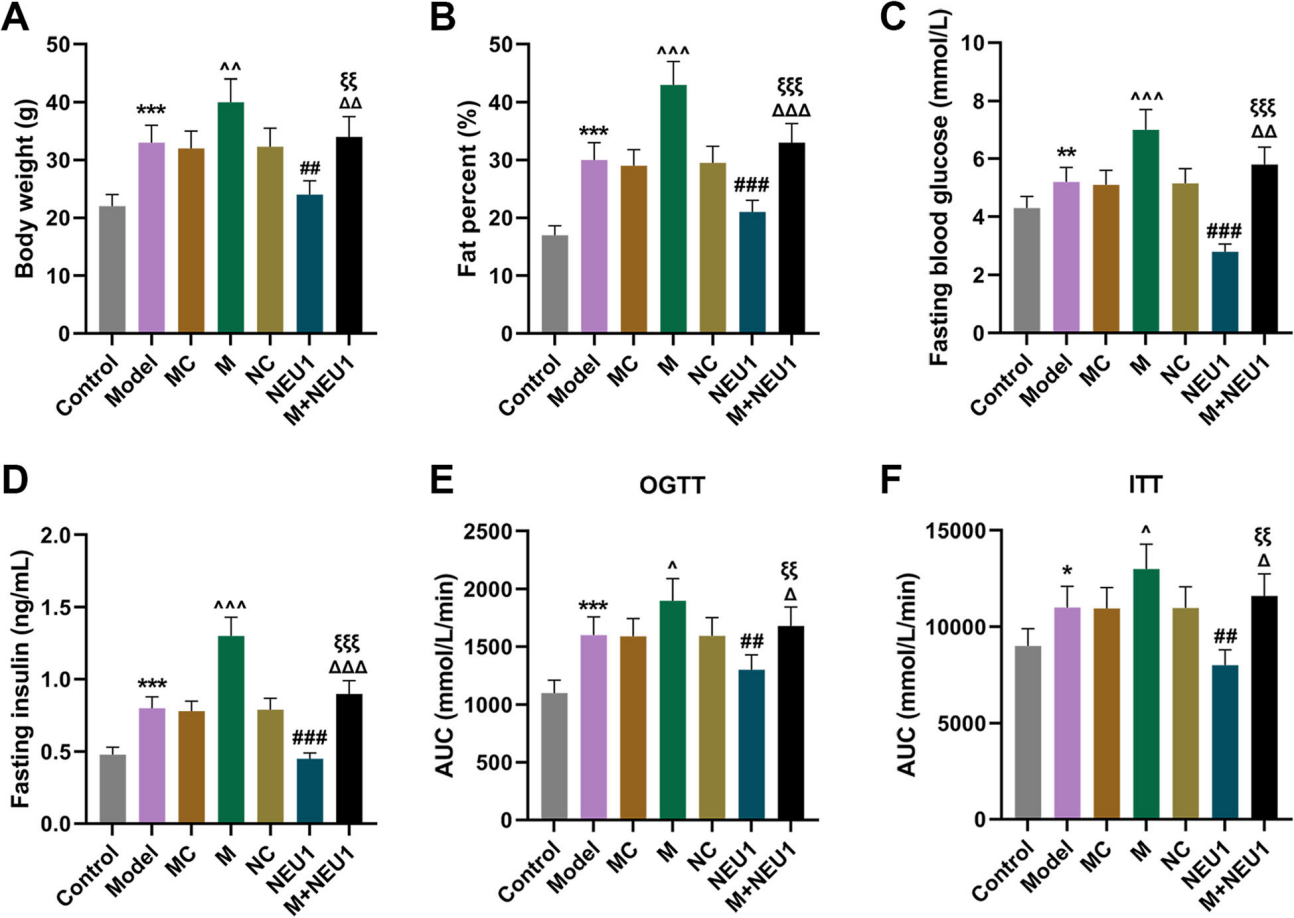
Overexpressed NEU1 reversed the effects of upregulated miR-23b-3p in obesity-induced IR of mice. **a** Body weight of mice after obesity-induced IR model construction and injection of lentivirus carriers for miR-23b-3p mimic and NEU1 overexpression plasmid was measured. **b** Fat percent of mice after obesity-induced IR model construction and injection of lentivirus carriers for miR-23b-3p mimic and NEU1 overexpression plasmid was calculated. **c-d** Levels of fasting blood glucose (**c**) and fasting insulin (**d**) after obesity-induced IR model construction and injection of lentivirus carriers for miR-23b-3p mimic and NEU1 overexpression plasmid were determined. **e-f** AUC of mice for OGTT (**e**) and ITT (**f**) after obesity-induced IR model construction and injection of lentivirus carriers for miR-23b-3p mimic and NEU1 overexpression plasmid was quantified. All experiments have been performed in triplicate and data were expressed as mean ± standard deviation (SD). ^*^P < 0.05, ^**^P < 0.01, ^***^P < 0.001, vs. Control; ^^^*P* < 0.05, ^^^^*P* < 0.01, ^^^^^*P* < 0.001, vs. MC; ^##^*P* < 0.01, ^###^*P* < 0.001, vs. NC; ^Δ^*P* < 0.05, ^ΔΔ^*P* < 0.01, ^ΔΔΔ^*P* < 0.001, vs. M; ^ξξ^*P* < 0.01, ^ξξξ^*P* < 0.001, vs. NEU1

To confirm the role and effects of NEU1 and miR-23b-3p on mice with obesity-induced IR, we first measured the body weight and fat percent of the mice following the model construction and injection of lentivirus carriers for miR-23b-3p mimic and NEU1 overexpression plasmid. In Fig. 4a-b, after model construction, both the body weight and fat percent of mice were increased (Fig. 4a-b, *P* < 0.001). Upregulating miR-23b-3p further enhanced the effects of obesity-induced IR on the body weight and fat percent of mice (Fig. 4a-b, *P* < 0.01). However, following NEU1 overexpression, the body weight and fat percent of the mice with obesity-induced IR were reduced, and overexpressed NEU1 could reverse the effects of upregulating miR-23b-3p (Fig. 4a-b, *P* < 0.01). Then we measured FBG and FI levels in mice. In Fig. 4c and d, it could be found that FBG and FI levels were elevated following model construction (Fig. 4c-d, *P* < 0.01). Upregulated miR-23b-3p mimic further upregulated FBG and FI levels (Fig. 4c-d, *P* < 0.001). After NEU1 overexpression, on the other hand, FBG and FI levels were downregulated (Fig. 4c-d, *P* < 0.001). Besides, NEU1 overexpression abrogated the effects of miR-23b-3p on FBG and FI levels in the mice with obesity-induced IR (Fig. 4c-d, *P* < 0.01).

Finally, to confirm the roles and effects of miR-23b-3p and NEU1 in mice with obesity-induced IR, we performed glucose and insulin tolerance tests. As shown in Fig. 4e and f, AUC of both OGTT and ITT was increased after the obesity-induced IR model construction (Fig. 4e-f, *P* < 0.05), and upregulated miR-23b-3p further increased AUC of OGTT and ITT (Fig. 4e-f, *P* < 0.05). We also found that overexpressed NEU1 reduced AUC of OGTT and ITT and reversed the effects of miR-23b-3p on the AUC of OGTT and ITT of obesity-induced IR mice (Fig. 4e-f, *P* < 0.05).

### Overexpressed NEU1 reversed the effects of upregulating miR-23b-3p on the levels of inflammatory cytokines and NEU1 expression

**Fig. 5:**
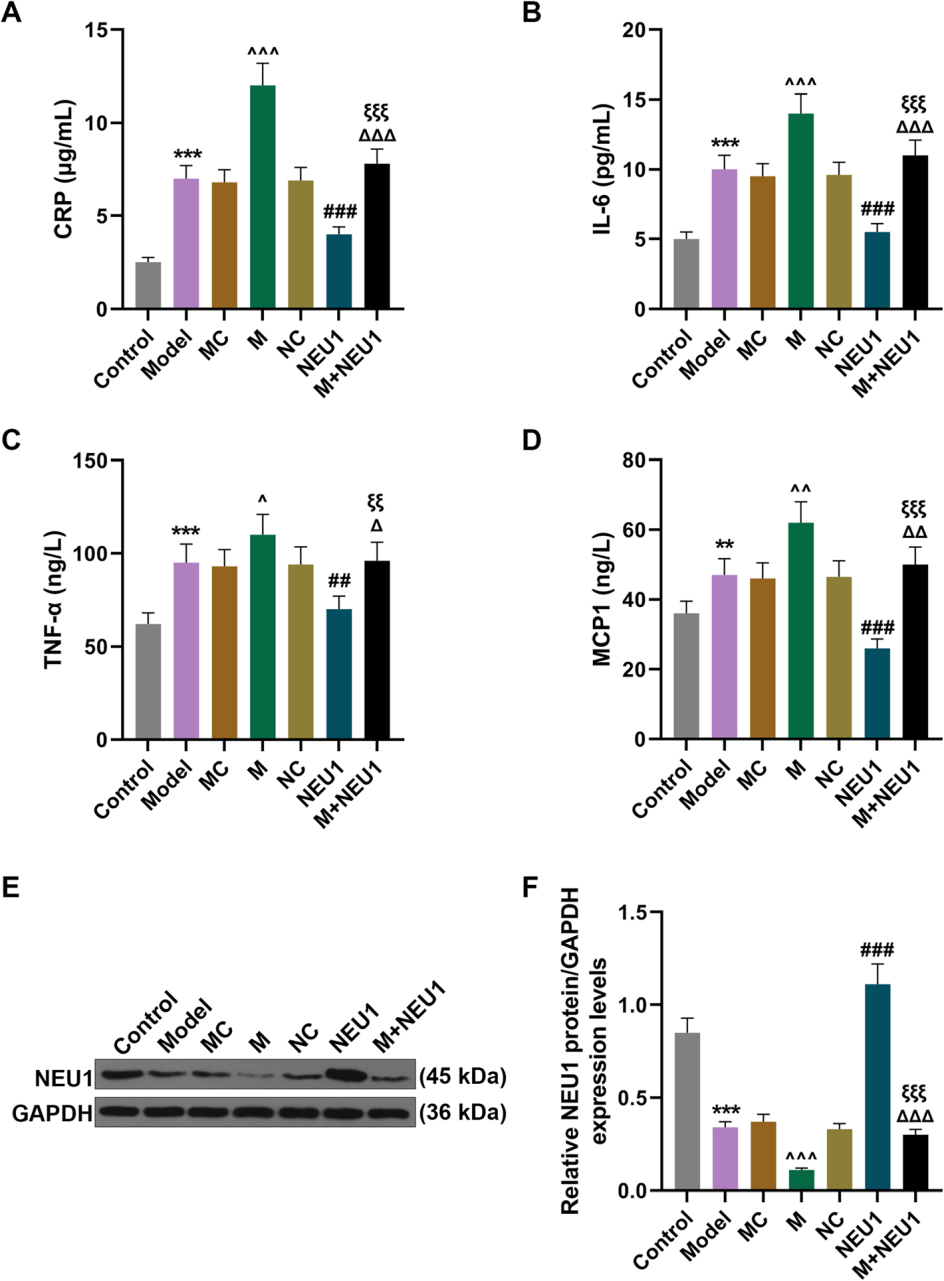
Overexpressed NEU1 reversed the effects of upregulated miR-23b-3p on inflammatory cytokines levels and NEU1 expression in obese mice. **a-d** Levels of inflammatory cytokines CRP (**a**), IL-6 (**b**), TNF-α (**c**) and MCP1 (**d**) after obesity-induced IR model construction and injection of lentivirus carriers for miR-23b-3p mimic and NEU1 overexpression plasmid were detected with ELISA. **e-f** NEU1 protein/GAPDH expressions after obesity-induced IR model construction and injection of lentivirus carriers for miR-23b-3p mimic and NEU1 overexpression plasmid were measured with Western blot. GAPDH was employed as internal control. All experiments have been performed in triplicate and data were expressed as mean ± standard deviation (SD). ^**^P < 0.01, ^***^P < 0.001, vs. Control; ^^^*P* < 0.05, ^^^^*P* < 0.01, ^^^^^*P* < 0.001, vs. MC; ^##^*P* < 0.01, ^###^*P* < 0.001, vs. NC; ^Δ^*P* < 0.05, ^ΔΔ^*P* < 0.01, ^ΔΔΔ^*P* < 0.001, vs. M; ^ξξ^*P* < 0.01, ^ξξξ^*P* < 0.001, vs. NEU1

To examine the possible effects of miR-23b-3p and NEU1 on mice, the levels of inflammatory cytokines (CRP, IL-6, TNF-α, and MCP) were measured with ELISA. In Fig. 5a-d, the levels of inflammatory cytokines were increased after the model was constructed (Fig. 5a-d,, *P* < 0.01). In addition, in obesity-induced IR mice, we observed that upregulating miR-23b-3p further enhanced the effects of obesity-induced IR on the levels of inflammatory cytokines, but overexpressed NEU1 reduced levels of inflammatory cytokines (Fig. 5a-d,, *P* < 0.05). Moreover, overexpressed NEU1 abrogated the functions of miR-23b-3p on inflammatory cytokines levels in obesity-mediated IR mice (Fig. 5a-d,, *P* < 0.05).

As shown in Fig. 5e-f, NEU1 protein expression was downregulated after model construction (Fig. 5e-f, *P* < 0.001 and additional file 1). Also, NEU1 protein expression was lower in the model mice after upregulating miR-23b-3p, but overexpressed NEU1 upregulated NEU1 protein expression (Fig. 5e-f, *P* < 0.001). We also found that NEU1 overexpression upregulated NEU1 protein expression and abrogated the effects of miR-23b-3p on NEU1 protein expression in the model mice (Fig. 5e-f, *P* < 0.001).

## Discussion

Previous studies showed that acacetin suppresses adipogenesis in 3 T3-L1 adipocytes and attenuates lipid accumulation in obesity mice [13]. MiR-23a-3p, a member of miR-23 family, plays a vital role in preventing against TNF-α-induced IR [20]. The plasma circulating miR-23b-3p at disease onset is associated with reduced insulin secretion in patients with type 1 diabetes [21]. The present findings suggested that acacetin treatment could improve obesity and control IR in obesity mice. However, upregulated miR-23b-3p reversed the effects of acacetin on mice with obesity-induced IR and chronic inflammation, and can further cause IR, ultimately leading to type 2 diabetes (T2D) [22]. Obesity-induced chronic inflammation has been detected in obesity-induced IR, and some inflammatory cytokines such as CRP, IL-6, TNF-α, and MCP1 are found in obesity-induced IR [23, 24]. In our current study, we observed that CRP, IL-6, TNF-α, and MCP1 levels were upregulated after the model construction. In addition, miR-23b-3p expression is upreulated in obesity-induced IR model, which is consistent with another report in which miR-23b-3p expression is promoted in soleus muscle of diet-induced obese (DIO) mice [25]. Acacetin exerts its anti-inflammatory effects on nucleus pulposus cells in tert-butyl peroxide (TBHP) condition [26], lipopolysaccharide (LPS)-stimulated human periodontal ligament (PDL) cells [27], sepsis-induced acute lung injury (ALI) [28]. In the present study, acacetin downregulated the levels of CRP, IL-6, TNF-α, MCP1, and miR-23b-3p. Moreover, upregulated miR-23b-3p reversed the effects above of acacetin on the mice with obesity-induced IR.

IR could lead to Treg/Th17 cell imbalance [29]. Balance of Treg/Th17 cells has long been the focus of some scientific studies aiming to examine the pathology of several autoimmune disorders [30]. Treg cells play a pivotal role in peripheral immune tolerance maintenance, and Th17 cells could cause autoimmunity and inflammation [31, 32]. Study showed that reducing the proportion of Treg cells lowers the inhibitory effects, resulting in enhanced activity of Th17 cells [33]. Previous study suggested that Treg/Th17 imbalance could contribute to the development of obesity [34]. Nevertheless, the detailed mechanisms in obesity-induced IR remained to be further addressed. MiRNAs have regulatory effects on Treg/Th17 balance in several autoimmune diseases [35]. The serum concentration of miR-23b-3p is associated with the immune and inflammatory progressions [36]. In our study, we found upregulated Th17 (IL-17) and downregulated Treg cell marker (Foxp3) in the model mice, suggesting that Treg/Th17 imbalance might also occur to obesity-induced IR mice. Moreover, upregulated miR-23b-3p reversed the effects of acacetin on downregulating IL-17 and upregulating Foxp3 in the mice with obesity-induced IR.

NEU1 is the most abundantly expressed sialidase in mammalians and has a catabolic function in lysosome. Overexpressed NEU1 could restore insulin signaling, and therefore might reverse IR [9, 37]. Previous study indicated that NEU1 may be the target of miR-125b [12], but the relationship between NEU1 and miR-23b-3p in obesity-mediated IR still needed to be addressed. In the present study, we confirmed that NEU1 was the target of miR-23b-3p, and that acacetin upregulated the NEU1 expression by downregulating miR-23b-3p expression in mice with obesity-induced IR.

In our present study, we found that after the construction of obesity-induced IR animal model, in total CD4^+^ T cells, Treg cells were reduced, Th17 cells were increased, levels of markers TGF-β1 and IL-10 were downregulated, and those of IL-17 and IL-6 were upregulated. These findings indicated that Treg/Th17 imbalance occurred during the development of obesity-induced IR. Moreover, these effects were further enhanced by miR-23b-3p upregulation. However, upregulating NEU1 reversed these effects of miR-23b-3p. Previous report showed that NEU1 regulates gene expression and secretion of IL-6 and MCP-1 via NF-kappaB pathway in 3 T3-L1 adipocytes [38]. NEU1 interacts with perilipin 1 on lipid droplets and suppresses lipolysis in 3 T3-L1 adipocytes [39]. In our study, upregulated NEU1 offset the effects of miR-23b-3p on body weight, fat percent, FBG, FI and AUC of both OGTT and ITT, and inflammatory factors (CRP, IL-6, TNF-α, MCP1) in mice with obesity-induced IR. Moreover, we also observed that miR-23b-3p upregulation repressed NEU1 expression in mice with obesity-induced IR, indicating that upregulated miR-23b-3p might enhance obesity-induced IR through regulating Treg/Th17 balance via targeting NEU1. Collectively, we demonstrated that acacetin ameliorates obesity-induced IR through regulating Treg/Th17 balance via miR-23b-3p/NEU1 axis.

## Conclusions

In conclusion, our study supports a new evidence of the role of acacetin in obesity-induced IR through regulating Treg/Th17 balance via targeting miR-23b-3p/NEU1 axis. The current results suggested that acacetin could be employed as a therapeutic drug to treat obesity-induced IR, and that acacetin appliance could serve as a novel method for treatment of obesity-related metabolism dysfunction.

## Supplementary Information

**Additional file 1.**

## Data Availability

The analyzed data sets generated during the study are available from the corresponding author on reasonable request.

## Abbreviations

miRs: microRNAs
IR: Insulin resistance
NEU1: Neuraminidase 1
CRP: C-reactive protein
IL-6: Interleukin-6
MCP1: Monocyte chemoattractant protein 1
ELISA: Enzyme-linked immunosorbent assay
qRT-PCR: Quantitative real-time polymerase chain reaction
BMI: Body mass index
HDL: High-density lipoprotein
ATMs: Adipose tissues
NEUs: Neuraminidases
ITT: Insulin tolerance test
BCA: Bicinchoninic acid
PVDF: Polyvinylidene fluoride
TBST: Tris-buffer saline tween
ECL: Enhanced chemiluminescence
