# Supplementary material for: Acacetin ameliorates insulin resistance in obesity mice through regulating Treg/Th17 balance via MiR-23b-3p/NEU1 Axis
Source: BMC Endocr Disord. 2021 Mar 29;21:57. doi: 10.1186/s12902-021-00688-8 (PMC8008644; doi:10.1186/s12902-021-00688-8)

Figure 3.E Protein/GAPDH expressions of TGF-β1, IL-10, IL-17 and IL-6 after obesity-induced IR model construction and injection of lentivirus carriers for miR-23b-3p mimic and NEU1 overexpression plasmid were measured with Western blot

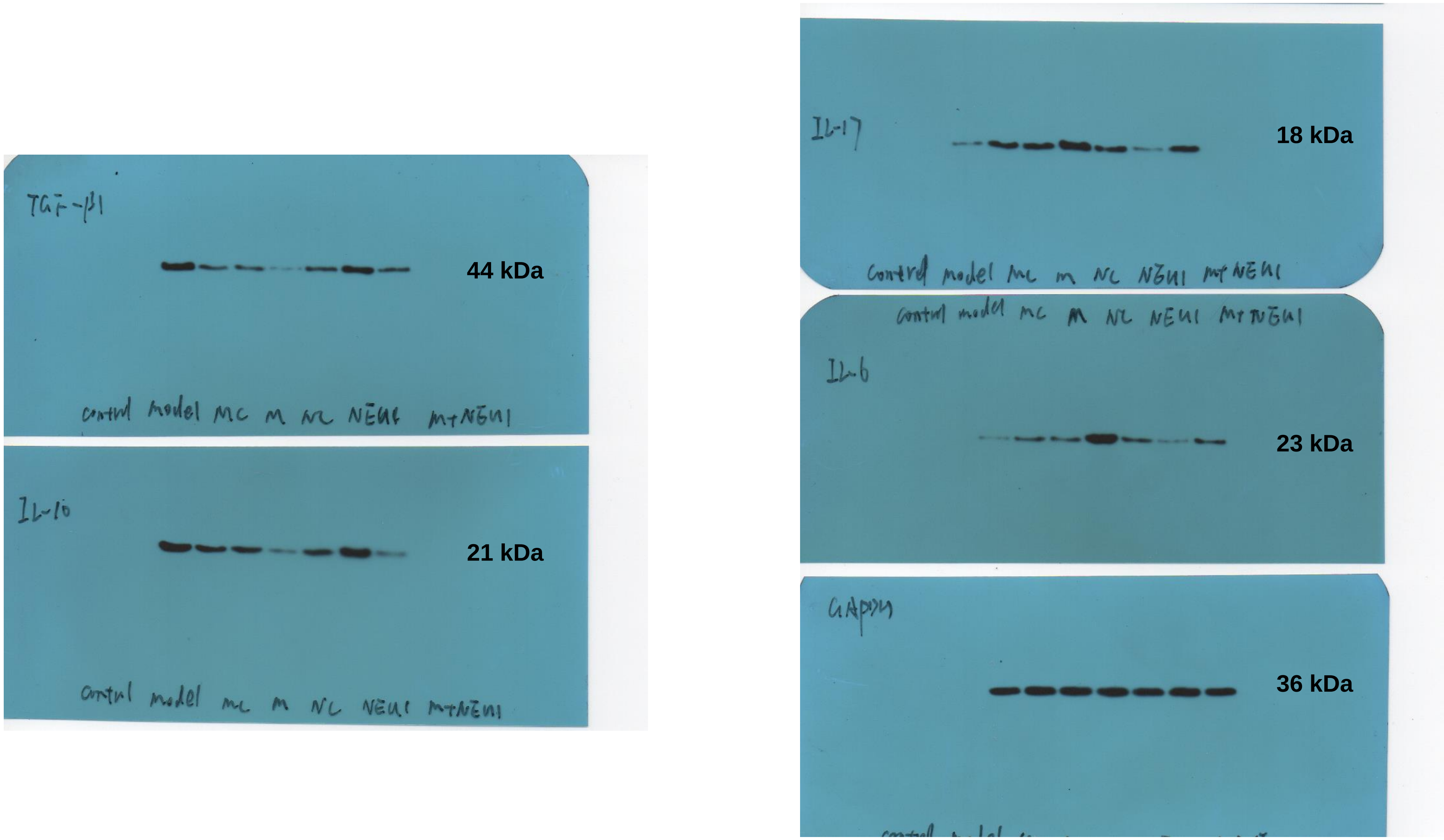

Figure 5.E NEU1 protein/GAPDH expressions after obesity-induced IR model construction and injection of lentivirus carriers for miR-23b-3p mimic and NEU1 overexpression plasmid were measured with Western blot.

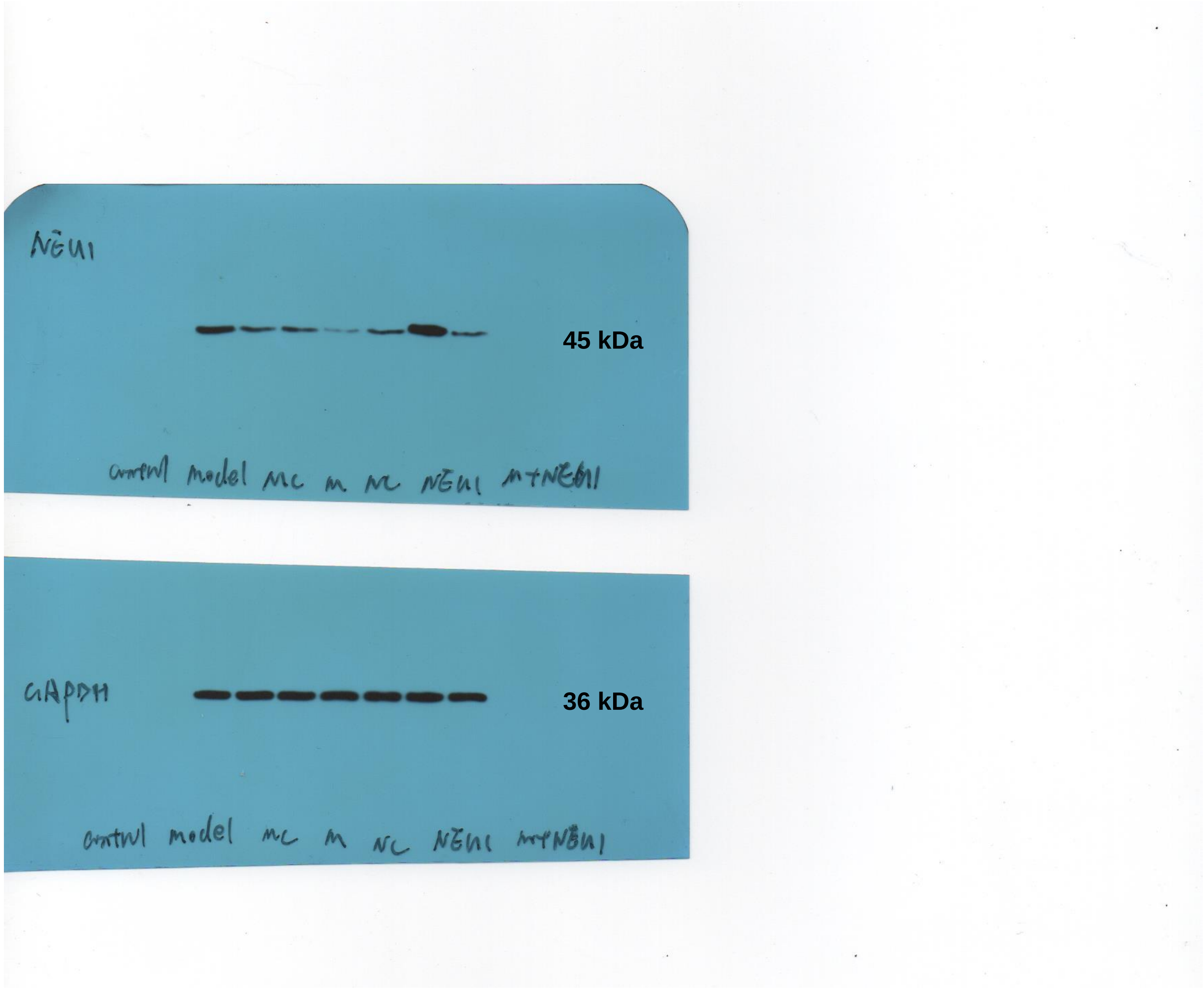

Supplement: Supplementary file 1 — Additional file 1. [file 12902_2021_688_MOESM1_ESM.pdf]
